# Proteasome inhibition induces a BRCAness-like state and sensitises HR-proficient ovarian cancer models to PARP inhibitors

**DOI:** 10.3389/fimmu.2026.1828316

**Published:** 2026-06-19

**Authors:** Ruiju He, Yingting Wei, Jing Niu, Jinghua Huang, Ruolan Wu, Kaiyan Gu, Anyue Wu, Lihua Qiu

**Affiliations:** 1 Department of Obstetrics and Gynaecology, Ren Ji Hospital, Shanghai Jiao Tong University School of Medicine, Shanghai, China; 2 Shanghai Key Laboratory of Gynaecologic Oncology, Ren Ji Hospital, Shanghai Jiao Tong University School of Medicine, Shanghai, China; 3 State Key Laboratory of Oncogenes and Related Genes, Shanghai Cancer Institute, Ren Ji Hospital, School of Medicine, Shanghai Jiao Tong University, Shanghai, China

**Keywords:** BRCAness-like state, homologous recombination proficient (HRP), immune recognition, ovarian cancer, PARP inhibitors, proteasome inhibitors

## Abstract

**Background:**

Poly (ADP-ribose) polymerase inhibitors (PARPi) have improved outcomes in ovarian cancer with homologous recombination deficiency (HRD). However, approximately half of high-grade serous ovarian cancers retain intact homologous recombination repair and are classified as homologous recombination–proficient (HRP), limiting the clinical benefit of PARPi in this population. Strategies capable of pharmacologically inducing a functional HRD-like state may expand the therapeutic scope of PARPi.

**Methods:**

A drug repurposing screen of 97 FDA-approved targeted agents was conducted in olaparib-insensitive HRP ovarian cancer cell models. Drug interactions were evaluated using cell viability and colony formation assays. Homologous recombination activity was assessed using RAD51 foci formation and DR-GFP reporter assays. DNA damage, cell cycle distribution, and apoptosis were analysed using immunofluorescence and flow cytometry. Antitumour efficacy was evaluated in xenograft models. Immune recognition-related features and susceptibility to CD8^+^ T-cell-mediated killing *in vitro* were assessed using peripheral blood mononuclear cell (PBMC) coculture assays.

**Results:**

Proteasome inhibitors emerged as the most consistent sensitising drug class. The oral proteasome inhibitor ixazomib citrate demonstrated robust synergy with olaparib across multiple HRP ovarian cancer models. Mechanistically, ixazomib citrate disrupted recruitment of the homologous recombination protein RAD51 to DNA damage sites without reducing its expression, thereby inducing a functional HRD-like state consistent with BRCAness. This disruption increased olaparib-induced DNA damage, resulting in G2/M arrest and apoptosis. In xenograft models, combination therapy significantly suppressed tumour growth compared with either agent alone. Proteasome inhibition also increased tumour-surface MHC class I expression and appeared to enhance susceptibility to CD8^+^ T-cell-mediated tumour cell killing *in vitro*.

**Conclusions:**

Ixazomib citrate sensitised HRP ovarian cancer models to PARP inhibition by functionally impairing homologous recombination repair. These findings provide preclinical support for proteasome inhibition as a PARPi-sensitising strategy in HRP ovarian cancer. The immune-related observations are based primarily on *in vitro* coculture experiments and should therefore be considered exploratory. Further validation in additional preclinical and clinically relevant models is warranted.

## Introduction

Ovarian cancer remains the most lethal gynaecological malignancy worldwide. Most patients are diagnosed with advanced-stage disease and initially respond to cytoreductive surgery followed by platinum-based chemotherapy. However, recurrence is common, and long-term survival remains poor, with five-year survival rates ranging from 10%–40% (1). New therapeutic strategies are therefore urgently needed to improve outcomes.

Poly (ADP-ribose) polymerase inhibitors (PARPi) have significantly altered the treatment landscape of ovarian cancer. Agents such as olaparib exploit synthetic lethality in tumours with defects in homologous recombination (HR) repair, particularly those harbouring mutations in BRCA1 or BRCA2 (2, 3). In this setting, PARP inhibition prevents repair of single-strand DNA breaks, leading to accumulation of double-strand breaks that cannot be effectively repaired in HR-deficient cells. This mechanism has translated into substantial improvements in progression-free survival in patients with homologous recombination deficiency (HRD) (4, 5).

Despite these advances, the clinical benefit of PARP inhibition remains limited in homologous recombination–proficient (HRP) ovarian cancer (6, 7). Approximately half of high-grade serous ovarian tumours retain intact homologous recombination repair capacity and are therefore classified as HRP (8, 9). In these tumours, efficient repair of DNA damage diminishes the cytotoxic effects of PARP inhibition, resulting in reduced therapeutic efficacy.

Beyond tumour-intrinsic DNA repair capacity, the tumour microenvironment may also contribute to treatment resistance. Recent transcriptomic analyses suggest that HRP tumours often exhibit an immune-cold or immune-excluded microenvironment characterised by limited cytotoxic T-cell infiltration and reduced immunogenicity (10, 11). Such features may further restrict the effectiveness of therapies that rely on DNA damage–induced immune activation.

Combination strategies aimed at inducing “BRCAness” have therefore been explored to extend the benefit of PARP inhibitors to HRP tumours. Various approaches targeting DNA damage response pathways, angiogenesis, or immune checkpoints have been investigated. However, large clinical trials evaluating several of these combinations have produced inconsistent results, indicating that additional approaches are needed (12, 13).

Drug repurposing offers a practical strategy to identify novel PARPi sensitising agents (14–16). Because repurposed drugs already possess established safety and pharmacokinetic profiles, they can be rapidly translated into clinical evaluation. In this study, we performed a systematic screen of FDA-approved targeted agents to identify compounds capable of sensitising HRP ovarian cancer cells to PARP inhibition.

Our screening identified proteasome inhibitors as a promising drug class, with ixazomib citrate demonstrating strong synergy with olaparib. We therefore investigated the molecular mechanisms underlying this interaction and evaluated its antitumour effects both *in vitro* and *in vivo*. In addition, we explored whether proteasome inhibition might modulate immune recognition-related features and alter the susceptibility of tumour cells to CD8^+^ T-cell-mediated killing *in vitro*.

## Materials and methods

### Cell culture

Human ovarian cancer cell lines with homologous recombination proficiency were cultured under standard conditions. Cells were maintained in RPMI-1640 medium supplemented with 10% foetal bovine serum and antibiotics at 37 °C in a humidified incubator containing 5% CO_2_.

### Drug screening

A library of 97 FDA-approved targeted agents was screened in HRP ovarian cancer cell models with intrinsic resistance to olaparib. Cells were treated with olaparib alone or in combination with individual compounds, and cell viability was measured after 72 hours using a luminescent viability assay. Drug interactions were quantified using combination index analysis.

### Cell viability and clonogenic assays

For viability assays, cells were seeded in 96-well plates and treated with drugs for 72 hours before measurement of metabolic activity. For colony formation assays, cells were treated with drugs for 24 hours and then cultured in drug-free medium for 10–14 days before fixation and staining. The working concentrations of ixazomib citrate and olaparib were selected based on dose–response assays in our preliminary experiments. For SKOV3 cells, the doses were 10 μM (olaparib) and 85 nM (ixazomib citrate); for ES2 cells, 5 μM and 60 nM; and for HEY cells, 5 μM and 70 nM, respectively, in all subsequent experiments.

### Homologous recombination assays

Homologous recombination activity was assessed using RAD51 foci formation following induction of DNA damage. Cells were exposed to ionising radiation or drug treatment and stained for RAD51 using immunofluorescence microscopy. A DR-GFP reporter assay was also used to measure homologous recombination repair efficiency (17, 18). For this assay, U2OS-DRGFP cells were treated with 5 μM olaparib, 10 nM ixazomib citrate, or their combination for 24 h prior to plasmid transfection.

### DNA damage and apoptosis analysis

DNA damage was quantified by immunofluorescence staining for γ-H2AX foci. Cell-cycle distribution was analysed by flow cytometry following propidium iodide staining. Apoptosis was assessed using Annexin V staining and caspase activation assays.

### Xenograft models

Female BALB/c nude mice (6 weeks old) were subcutaneously injected with 5 × 10^6^ SKOV3 cells. Once tumours reached a mean diameter of 3–4 mm (approximately 30 mm³ volume), animals were randomised into four groups (n = 5 per group) to receive vehicle, olaparib, ixazomib citrate, or their combination. Drugs were administered by oral gavage for 3 weeks: ixazomib citrate at 5 mg/kg twice weekly, olaparib at 40 mg/kg daily (5 days/week), and the combination group received both agents on the same schedule. Tumour volume was measured twice weekly using calipers and calculated with the formula: Volume = (Length × Width²)/2. After three weeks, all mice were euthanized. Excised tumour tissues were fixed, paraffin-embedded and sectioned for immunohistochemical staining. The expression levels of Ki-67 and cleaved caspase-3 in tumour tissues were detected following standard IHC protocols.

### Immune coculture assays

Tumour cells were cocultured with human peripheral blood mononuclear cells (PBMCs) from healthy donors. Expression of MHC class I molecules on tumour cells and markers of CD8^+^ T-cell activation were assessed using flow cytometry. For CD8^+^ T-cell analysis, lymphocytes were first gated by FSC-A/SSC-A, followed by singlet discrimination using FSC-A/FSC-H. Live CD45^+^ leukocytes were selected based on viability dye and CD45 staining. CD3^+^CD8^+^ T cells were then identified, and IFN-γ expression was assessed using standard gating strategies. Cytotoxicity assays were performed to evaluate tumour cell susceptibility to T-cell–mediated killing.

### Statistical analysis

Data are presented as mean ± standard deviation. Statistical comparisons were performed using Student’s t-test or one-way ANOVA followed by Tukey’s multiple comparisons test. where appropriate. A P value <0.05 was considered statistically significant.

## Results

### Drug screening identifies proteasome inhibitors as PARP inhibitor sensitisers

**Figure 1:**
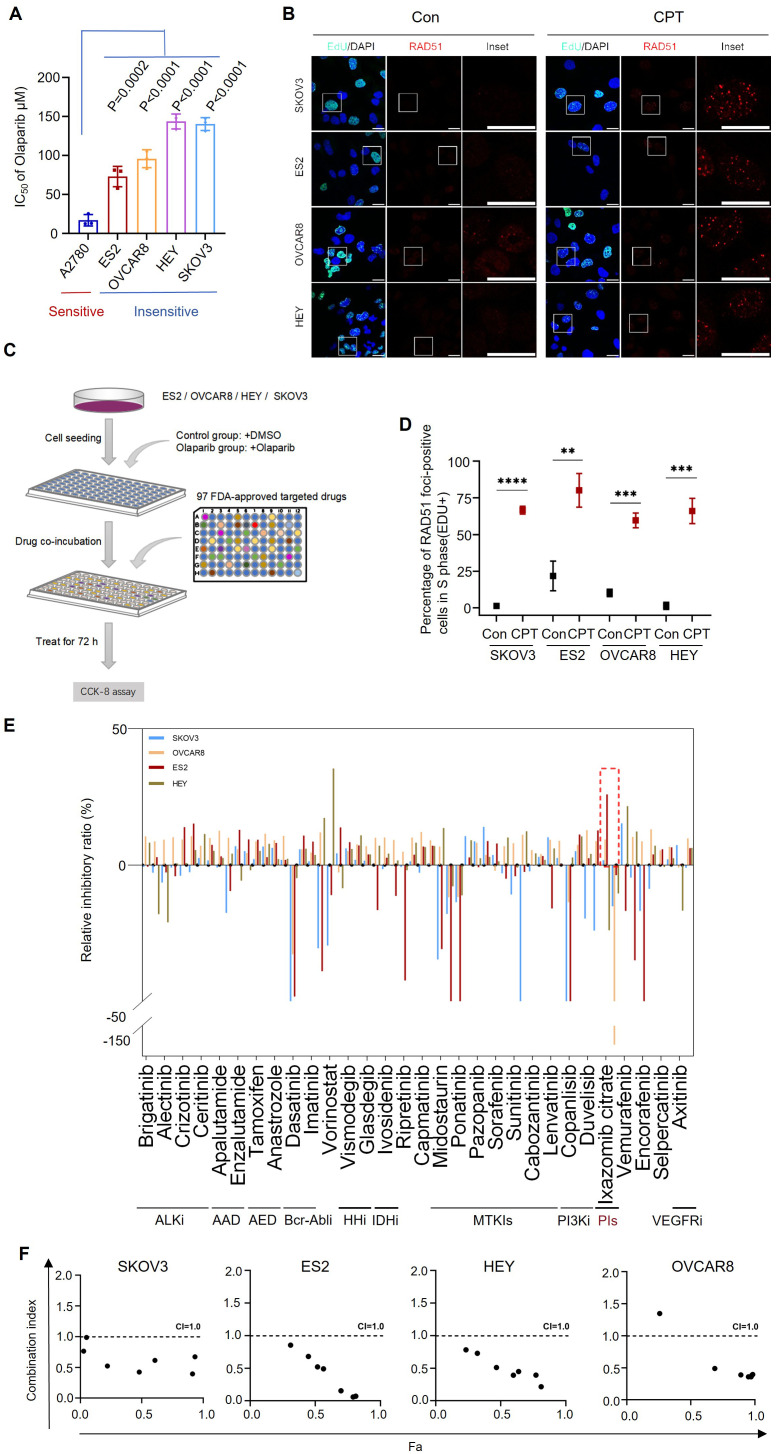
FDA-approved drug screening identifies ixazomib citrate as a synergistic partner of olaparib inHRP ovarian cancer. **(A)** IC_50_ values of Olaparib after 72 h treatment ina panel of ovarian cancer cell lines, classifying A2780 as olaparib-sensitive and ES2, OVCAR8, HEY, SKOV3 as olaparib-insensitive. **(B)** Representative immunofluorescence images showing EdU (green, S-phase marker), RAD51 (red, HR repair marker), and DAPI (blue, nuclear counterstain) after camptothecin (CPT,100 nM) treatment for 2 hours; cells with >5 RAD51 foci per nucleus were defined as RAD51 foci-positive.scale bar, 20 μm. **(C)** Schematic of the high-throughput drug screening workflow: 97 FDA-approved targeted drugs were tested in combination with olaparib for 72 h, and cell viability was measured using the CCK-8 assay. **(D)** Quantification of RAD51 foci-positive cells among EdU^+^ cells, confirming robust homologous recombination repair capacity in the four olaparib-insensitive cell lines. **(E)** Screening results showing relative inhibitory ratios. Ixazomib citrate (red box) was identified as the top candidate with the strongest synergy. **(F)** Combination index (CI) values at different fractional effects (Fa), calculated using the Chou-Talalay method. CI < 1 indicates synergistic interaction between ixazomib citrate and olaparib. Data are presented as mean ± SD, n = 3 independent experiments; two-tailed unpaired Student’s t-test. *p < 0.05, **p < 0.01, ****p < 0.0001.7.

To identify agents capable of sensitising homologous recombination proficient (HRP) ovarian cancer to PARP inhibition, we first determined olaparib IC_50_ values in a panel of ovarian cancer cell lines. SKOV3, ES2, OVCAR−8 and HEY were confirmed to be olaparib−insensitive, in contrast to the sensitive line A2780 (**Figure 1A**). These lines showed robust homologous recombination repair, with >50% of cells forming RAD51 foci after CPT (camptothecin)-induced DNA damage (**Figures 1B, D**; **Supplementary Figure S1A**), and were thus defined as HRP models.

Screening of 97 FDA−approved targeted drugs identified proteasome inhibitors as the most consistent enhancers of olaparib cytotoxicity across cell lines (**Figures 1C, E**). Among them, ixazomib citrate produced the most reproducible synergy with olaparib: combination treatment reduced cell viability and colony formation substantially more effectively than single−agent therapy (**Figures 1F**; **Supplementary Figures S1B−I**).

### Proteasome inhibition induces a functional HRD-like state

**Figure 2:**
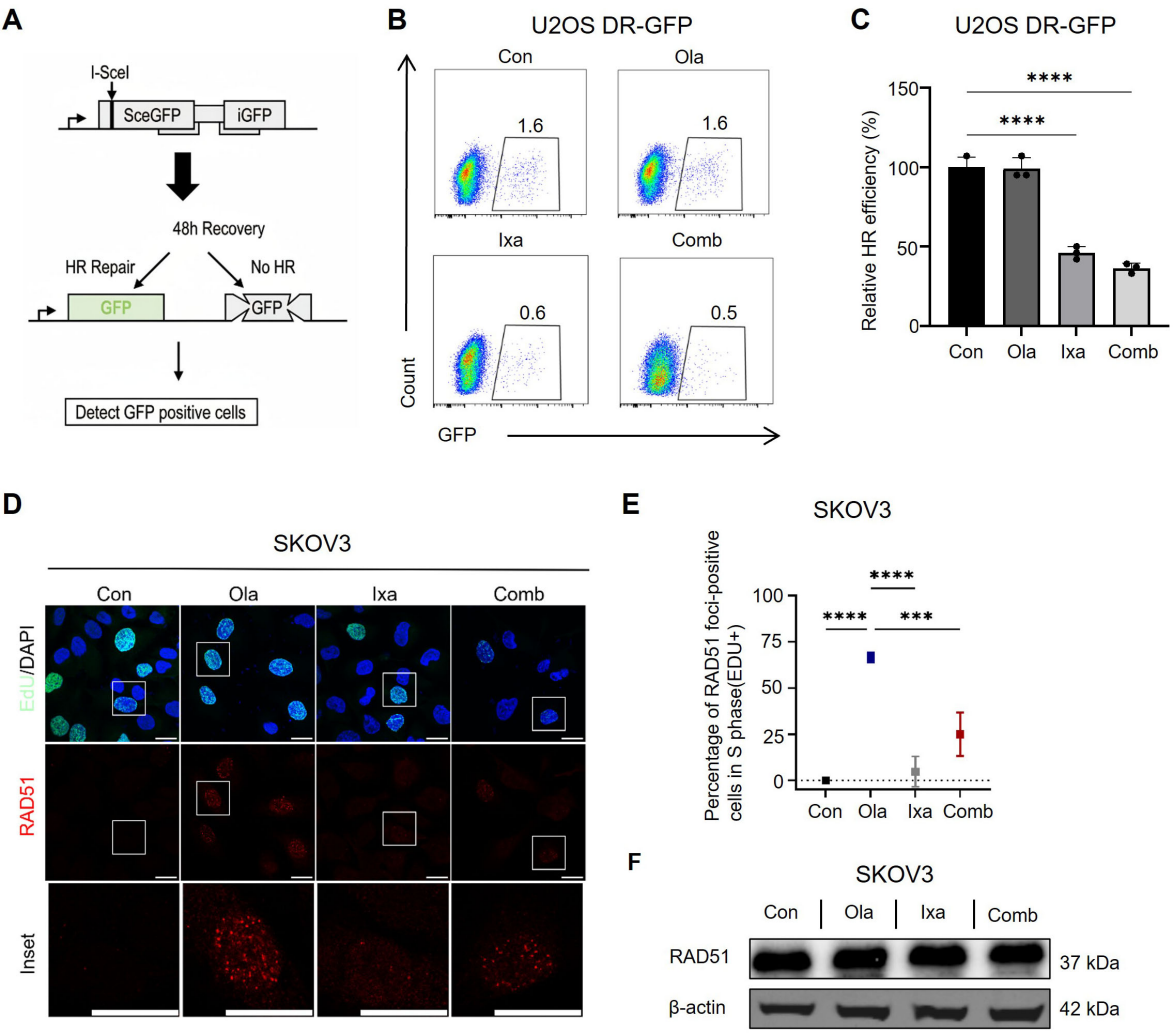
Ixazomib citrate, alone or in combination with olaparib, impairs homologous recombination repair. **(A)** Schematic diagram of the DR-GFP reporter system applied to detect cellular homologous recombination (HR) repair efficiency following I-SceI endonuclease-induced DNA double-strand breaks (DSBs). **(B, C)** Representative flow cytometry plots and corresponding quantitative analysis of GFP-positive cells in PI-negative viable U2OS DR-GFP reporter cells, reflecting the changes in intracellular HR repair efficiency after different drug interventions. **(D, E)** Representative immunofluorescence images of RAD51 foci formation and statistical analysis. The proportion of EdU-positive proliferative SKOV3 cells carrying more than 5 RAD51 foci was calculated to evaluate the recruitment ability of RAD51 towards DNA damage sites. Scale bar, 20 μm. **(F)** Western blot analysis showing the total protein expression level of RAD51, with β-actin served as the internal reference protein for loading normalization. Data are presented as mean ± SD, n = 3 independent experiments; Statistical differences were analyzed via one-way ANOVA followed by Tukey’s multiple comparisons test. ***p < 0.001, ****p < 0.0001.

To investigate the mechanism underlying this synergy, we examined homologous recombination repair activity. Treatment with ixazomib citrate alone did not substantially alter the protein expression level of RAD51(**Figure 2F**; **Supplementary Figures 2B**), nor did it affect the mRNA expression of BRCA1, BRCA2, RAD51 and PALB2 (**Supplementary Figures 2A**). However, it significantly impaired recruitment of RAD51 to sites of DNA damage (**Figures 2D, E**). Consistent with this observation, DR-GFP reporter assays demonstrated reduced HR repair efficiency following proteasome inhibition (**Figures 2A–C**).

In contrast, ixazomib citrate did not affect the non-homologous end joining (NHEJ) pathway: neither the formation of 53BP1 foci nor the total protein level of 53BP1 were altered by ixazomib treatment (**Supplementary Figures 2C–E**). Together, these findings indicate that ixazomib citrate selectively suppresses HR repair function, inducing a functional HRD-like state without directly inhibiting the expression of core HR proteins, thereby creating a BRCAness-like state.

### Combination treatment increases DNA damage and apoptosis

**Figure 3:**
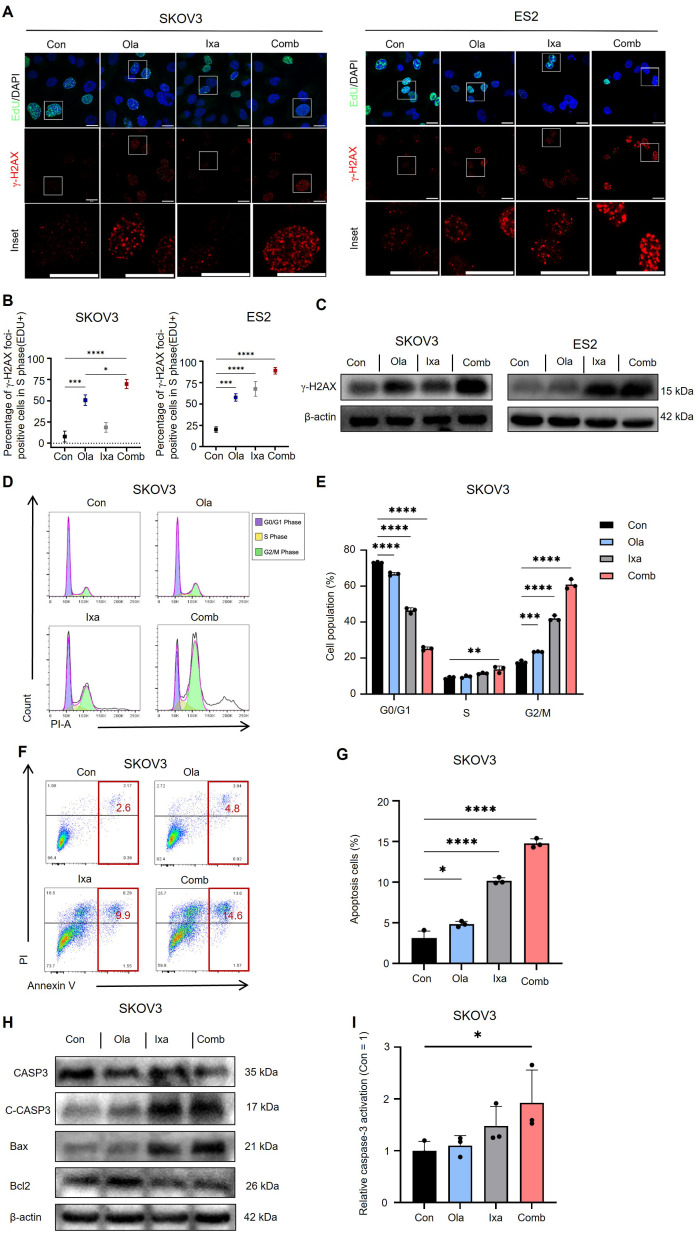
Ixazomib citrate potentiates olaparib-induced G2/M arrest and apoptosis. **(A)** Representative immunofluorescence images showing γ-H2AX (red, a specific marker of DNA double-strand breaks), and EdU (green) in SKOV3 (Ola 10 μM, Ixa 85 nM) and ES2 (Ola 5 μM, Ixa 60 nM); DAPI (blue); scale bar, 20 μm. **(B)** Quantification of EdU^+^ S-phase cells containing >10 γ-H2AX foci per cell, used to evaluate the extent of DNA damage accumulation in proliferating cells after different drug treatments. **(C)** Western blot of γ−H2AX protein levels; β-actin as loading control. **(D, E)** Representative flow cytometry plots **(D)** and corresponding quantitative analysis **(E)** of cell cycle distribution (G0/G1, S, G2/M) in SKOV3 cells after 48 hours of treatment with vehicle control (Con), olaparib (Ola) alone, ixazomib citrate (Ixa) alone, or their combination. **(F, G)** Annexin V/PI flow cytometry plots **(F)** and quantification **(G)** of apoptotic cells after 72 h treatment. **(H, I)** Western blot analysis of Bcl−2, Bax, total caspase−3 (CASP3) and cleaved caspase−3 (C−CASP3). The C−CASP3/CASP3 ratio was quantified and normalized to the control group (set as 1). β−actin served as loading control. Data are presented as mean ± SD, n = 3 independent experiments; Statistical differences were analyzed via one-way ANOVA. *p < 0.05, **p < 0.01, ***p < 0.001, ****p < 0.0001.

We next examined whether impaired homologous recombination enhanced the cytotoxic effects of PARP inhibition. Ixazomib citrate alone significantly increased γ-H2AX foci accumulation, and this effect was further enhanced by combination with olaparib, indicating elevated DNA double-strand break formation (**Figures 3A–C**).

Flow cytometric analysis revealed pronounced G2/M cell-cycle arrest following combination therapy (**Figures 3D, E**). This was accompanied by increased Annexin V staining and caspase activation, indicating enhanced apoptotic cell death (**Figures 3F, G**). Consistently, Western blotting showed the combination significantly upregulated cleaved caspase-3 (C-CASP3) and pro-apoptotic Bax, while downregulating anti-apoptotic Bcl-2 (**Figure 3H**). Quantification of the C-CASP3/CASP3 ratio confirmed enhanced caspase-3 activation in the combination group (**Figure 3I**).

### Combination therapy suppresses tumour growth *in vivo*

**Figure 4:**
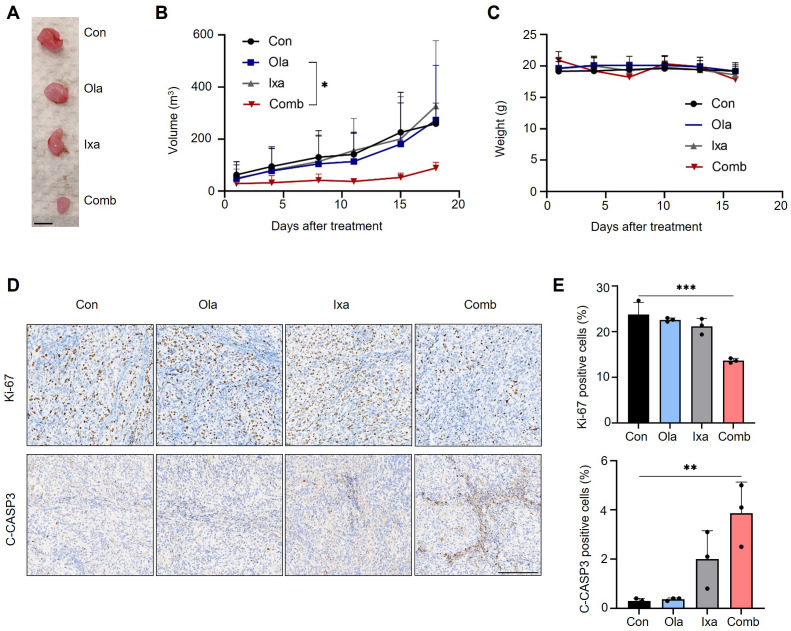
Combined olaparib and ixazomib citrate suppress SKOV3 xenograft growth *in vivo*. **(A)** Representative photographs of excised tumours from each treatment group after 3 weeks of drug administration; scale bar, 5 mm. **(B)** Tumour growth curves showing the dynamic changes in tumour volume of mice in each group during the 3-week treatment period., n=5. **(C)** Mouse body weight during treatment. **(D, E)** Representative immunohistochemical (IHC) staining images **(D)** and corresponding quantitative analysis of positive cells **(E)** for Ki-67 (cell proliferation marker) and cleaved caspase-3 (C-CASP3, apoptotic marker) in tumour tissues. I scale bar, 200µm. Data are presented as mean ± SD, n = 3 independent experiments; Statistical differences were analyzed via one-way ANOVA. *p < 0.05, **p < 0.01, ***p < 0.001.

The therapeutic potential of this combination was evaluated in SKOV3 ovarian cancer xenograft models. Treatment with either olaparib or ixazomib citrate alone modestly inhibited tumour growth. In contrast, the combination produced a significantly greater reduction in tumour volume (**Figures 4A, B**). Importantly, no significant weight loss or overt toxicity observed in treated animals (**Figures 4C**). Immunohistochemical analysis of tumour tissues showed downregulated Ki-67 and upregulated cleaved caspase-3 in the combined treatment group (**Figures 4D, E**), which was consistent with our *in vitro* results.

### Proteasome inhibition increases immune recognition-related features *in vitro*

**Figure 5:**
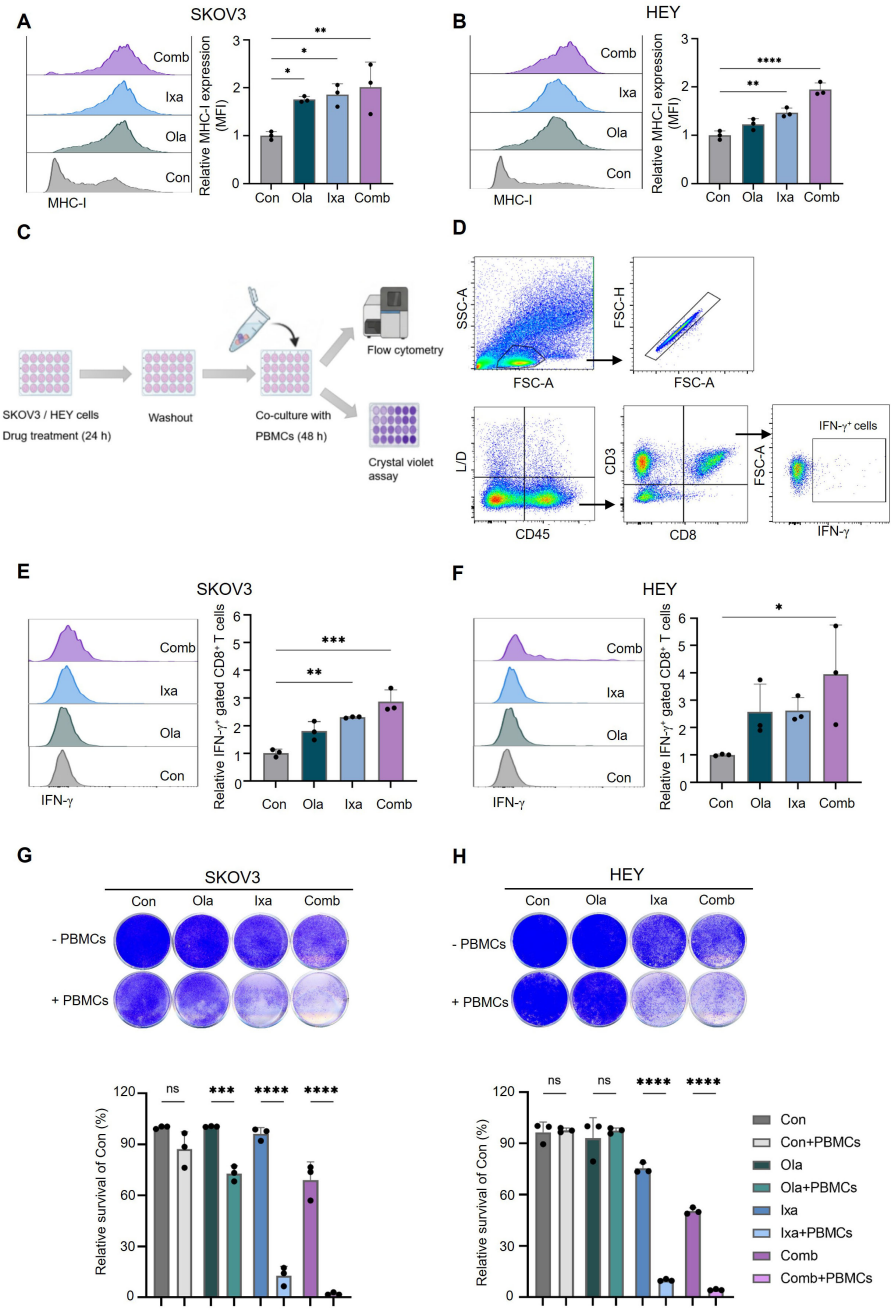
Olaparib–ixazomib citrate combination enhances immune recognition-related features and susceptibility to CD8^+^ T-cell-mediated killing *in vitro*. **(A)** Flow cytometry analysis of MHC−I expression on SKOV3 cells following 24 h treatment with vehicle (Con), olaparib (Ola, 10 μM), ixazomib citrate (Ixa, 85 nM), or their combination (Comb). Y-axis: Relative MHC−I expression (mean fluorescence intensity, MFI), normalized to the untreated control group (Con = 1). **(B)** Flow cytometry analysis of MHC−I expression on SKOV3 cells following 24 h treatment with vehicle (Con), olaparib (Ola, 5 μM), ixazomib citrate (Ixa, 70 nM), or their combination (Comb). **(C)** Co-culture workflow: drug-pretreated tumour cells ± PBMCs (1:5) for 48 h, followed by flow cytometry or crystal violet staining. **(D)** Flow cytometry gating strategy for IFN-γ^+^ CD8^+^ T cells. **(E, F)** Representative flow cytometry analysis and quantification of IFN-γ^+^ CD8^+^ T-cells after co-culture with SKOV3 **(E)** or HEY **(F)** cells; results were normalized to the control group. **(G, H)** Tumour cell survival rate in the presence or absence of PBMCs, quantified by crystal violet staining after 48 h of co-culture. Data are presented as mean ± SD, n = 3 independent experiments; one-way ANOVA. *p < 0.05, **p < 0.01, ***p < 0.001,****p < 0.0001.

Given the emerging link between DNA damage and tumour immune activation, we next examined whether proteasome inhibition affects tumour cell immune recognition and T−cell−mediated killing *in vitro*. Ixazomib citrate, alone or with olaparib, upregulated surface MHC−I expression on SKOV3 and HEY cells (**Figure 5A, B**). In PBMC coculture assays (**Figure 5C**), CD8^+^ T cells exposed to ixazomib−pretreated tumour cells showed increased CD25 activation, IFN−γ and granzyme B production (**Figures 5D–F**; **Supplementary Figure 3A–C**). Consequently, these tumour cells became more susceptible to PBMC−mediated killing (**Figures 5G, H**). Together, these findings indicate that proteasome inhibition alters tumour cell features associated with immune recognition *in vitro*.

## Discussion

PARP inhibitors have transformed the treatment of ovarian cancer with homologous recombination deficiency (HRD). However, their clinical impact remains limited in homologous recombination–proficient (HRP) disease. Developing strategies to extend PARPi sensitivity to HRP tumours therefore represents an important therapeutic goal.

In this study, we performed a systematic drug repurposing screen to identify agents capable of sensitising HRP ovarian cancer cells to PARP inhibition. Proteasome inhibitors emerged as a highly promising class, with ixazomib citrate demonstrating robust synergy with olaparib across multiple experimental systems.

Mechanistically, our findings indicate that proteasome inhibition disrupts homologous recombination (HR) repair by impairing recruitment of RAD51 to DNA damage sites. Importantly, this effect occurred without substantial changes in the expression of key HR proteins, suggesting that proteasome inhibition induces a functional rather than transcriptional HR defect. Taken together, these data support the interpretation that proteasome inhibition induces a functional HRD-like state consistent with BRCAness in the preclinical models examined. This phenomenon resembles the concept of pharmacological “BRCAness,” in which HRP tumours acquire characteristics of HRD and thereby become susceptible to PARP inhibition (19).

The combination of ixazomib citrate and olaparib resulted in marked accumulation of DNA damage, cell-cycle arrest, and apoptosis in HRP ovarian cancer cells (20). These findings were supported by *in vivo* studies demonstrating significant tumour growth inhibition with combination therapy.

In addition to its effects on DNA repair, proteasome inhibition appeared to modulate immune recognition-related tumour cell features *in vitro*. Increased MHC class I expression and enhanced CD8^+^ T-cell–mediated cytotoxicity was observed following ixazomib citrate treatment. DNA damage induced by PARP inhibition has previously been shown to activate innate immune signalling pathways (21, 22), and our findings raise the possibility that proteasome inhibition may further influence these processes (23). These observations suggest that drug-treated tumour cells may become more susceptible to immune recognition or CD8^+^ T-cell–mediated killing, although the *in vivo* relevance of this effect remains to be established.

Several limitations should be acknowledged. First, our study lacked validation in primary patient-derived samples or more clinically relevant models. Second, the PBMC co-culture assays employed PBMCs from healthy donors, resulting in allogeneic HLA mismatch, which does not recapitulate the autologous T-cell responses typical of the tumour microenvironment. Third, the heterogeneity of HRP ovarian cancer may limit the generalisability of our findings. Fourth, the precise mechanisms by which proteasome inhibition impairs RAD51 recruitment remain to be elucidated. In addition, caspase-3 activation in xenograft tumours was only assessed by IHC, as fresh-frozen tissue was not available for Western blot validation; we plan to include such protein-level validation, as well as comprehensive biosafety assessments (including major organ histology and serum biochemistry), in future studies.

Despite these limitations, our results provide preclinical support for the concept that proteasome inhibition may induce a BRCAness-like state and enhance sensitivity to PARP inhibition in HRP ovarian cancer models. Further studies in additional preclinical and clinically relevant systems will be required to define the scope and translational potential of this strategy.

## Conclusions

Proteasome inhibition may represent a promising PARPi-sensitising strategy in HR-proficient ovarian cancer models. The proteasome inhibitor ixazomib citrate induces a functional HRD-like state consistent with BRCAness, enhances olaparib-induced DNA damage, and increases tumour cell susceptibility to immune recognition *in vitro*. These findings provide a preclinical rationale for evaluating this combination as a therapeutic approach to expand the benefit of PARP inhibitors in HRP ovarian cancer. Additional validation in broader preclinical systems and clinically relevant models will be necessary prior to clinical application in patients with HRP ovarian cancer.

## Data Availability

The original contributions presented in the study are included in the article/**Supplementary Material**. Further inquiries can be directed to the corresponding authors.

## Supplementary material

Supplementary Figure 1 Ixazomib citrate synergizes with olaparib in HRP ovarian cancer cells. **(A)** Quantification of EdU^+^ S-phase cells with >10 γ-H2AX foci per cell, following 2 h treatment with camptothecin (CPT, 100 nM). **(B, D, F, H)** Dose–response curves of SKOV3, ES2, HEY, and OVCAR8 cells after 72 h treatment with olaparib, ixazomib citrate, or their combination. **(C, E, G, I)** Representative colony formation images (left) and Bliss synergy score heatmaps (right) for SKOV3, ES2, HEY, and OVCAR8 cells treated with serial dilutions of olaparib and ixazomib citrate alone or in combination for 5 days. Bliss scores > 0 indicate synergy (red = stronger synergy; grey = antagonism). Data are mean ± SD from three independent experiments. ***p < 0.001, **** < 0.0001.

Supplementary Figure 2 Ixazomib citrate minimally affects Rad51 or 53BP1 in SKOV3. **(A)** RT–qPCR of HR genes (BRCA1, BRCA2, RAD51, PALB2) in SKOV3 cells treated with ixazomib citrate (85 nM) for 0–24 h. mRNA levels were normalized to ACTB. **(B)** Immunoblot analysis of RAD51 protein expression in SKOV3 cells treated with ixazomib citrate (85 nM) for 0–24 h; β-actin served as the loading control. **(C)** Representative immunofluorescence images of 53BP1 foci (red) in SKOV3 cells after 24 h treatment with vehicle, olaparib (10 μM), ixazomib citrate (85 nM), or their combination. Nuclei were counterstained with DAPI (blue); scale bar, 20 μm. **(D)** Quantification of cells with >3 53BP1 foci per nucleus, calculated from ≥50 cells per condition. **(E)** Immunoblot analysis of 53BP1 protein levels in SKOV3 cells under the indicated treatments; β-actin served as the loading control. Data mean ± SD, n = 3; one-way ANOVA.

Supplementary Figure 3 Combined treatment enhances CD8^+^ T-cell activation. **(A)** Relative percentage of CD25^+^ cells among CD8^+^ T cells after co-culture with drug-pretreated SKOV3 or HEY cells, normalized to the control group. **(B, C)** Representative histograms **(B)** and quantification **(C)** of granzyme B (GZMB) expression in CD8^+^ T cells following co-culture with drug-pretreated SKOV3 or HEY cells. Data mean ± SD, n = 3; one-way ANOVA. *p < 0.05, **p < 0.01.
