# Supplementary figures and images for: Proteasome inhibition induces a BRCAness-like state and sensitises HR-proficient ovarian cancer models to PARP inhibitors
Source: Front Immunol. 2026 Jun 19;17:1828316. doi: 10.3389/fimmu.2026.1828316 (PMC13328278; doi:10.3389/fimmu.2026.1828316)

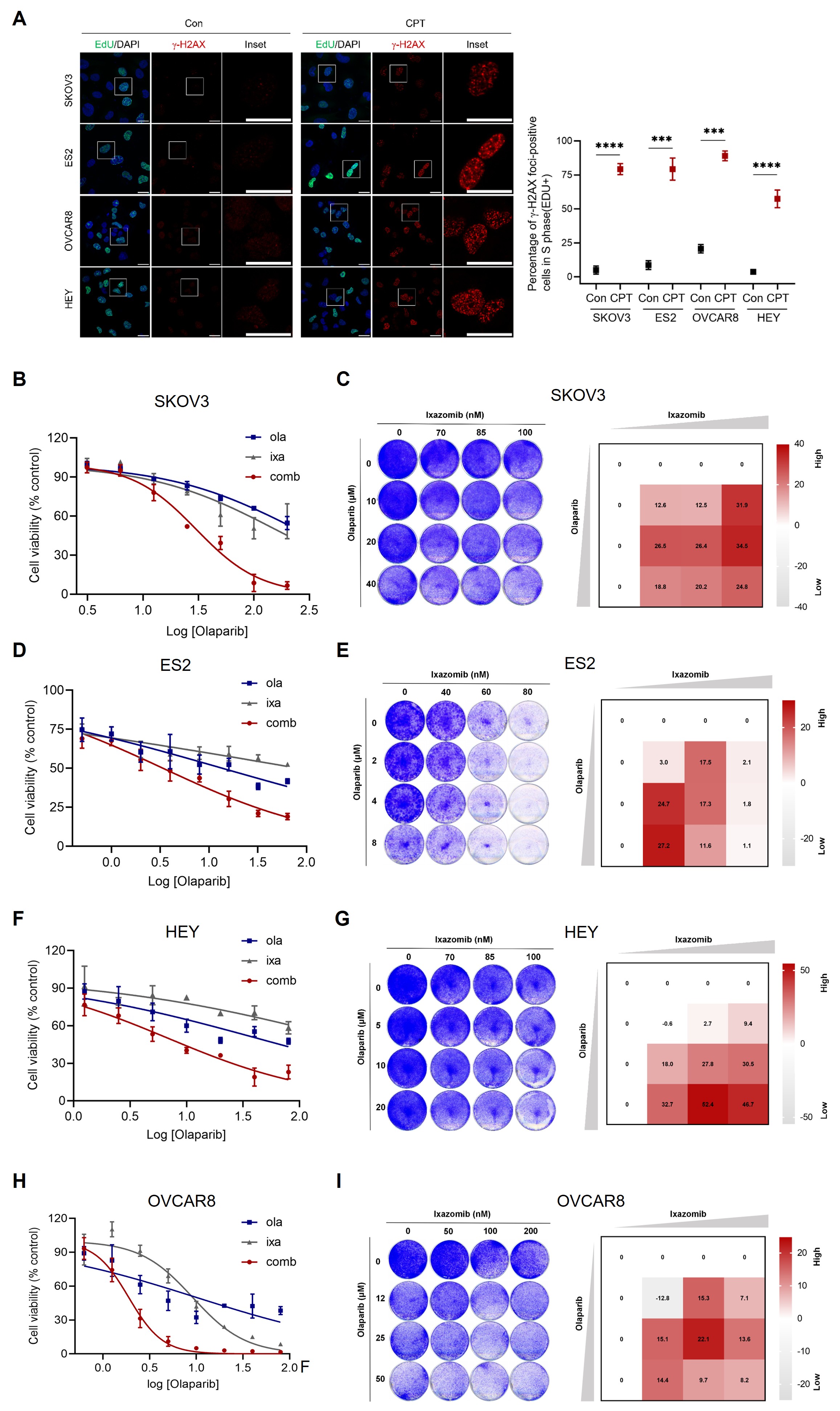

Supplement: Supplementary Figure 1 — Ixazomib citrate synergizes with olaparib in HRP ovarian cancer cells. (A) Quantification of EdU+ S-phase cells with >10 γ-H2AX foci per cell, following 2 h treatment with camptothecin (CPT, 100 nM). (B, D, F, H) Dose–response curves of SKOV3, ES2, HEY, and OVCAR8 cells after 72 h treatment with olaparib, ixazomib citrate, or their combination. (C, E, G, I) Representative colony formation images (left) and Bliss synergy score heatmaps (right) for SKOV3, ES2, HEY, and OVCAR8 cells treated with serial dilutions of olaparib and ixazomib citrate alone or in combination for 5 days. Bliss scores > 0 indicate synergy (red = stronger synergy; grey = antagonism). Data are mean ± SD from three independent experiments. ***p < 0.001, **** < 0.0001. [file Image1.jpeg]

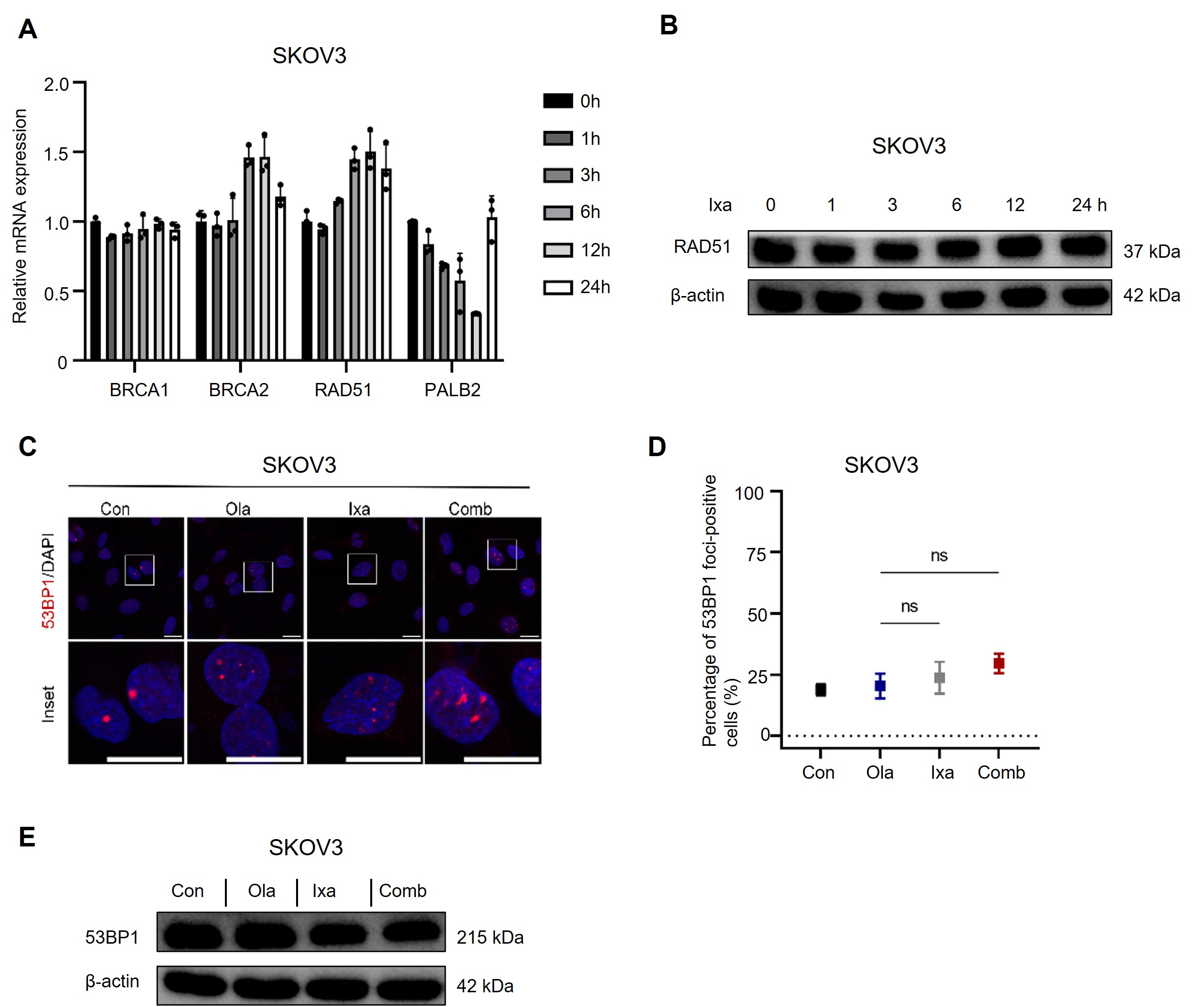

Supplement: Supplementary Figure 2 — Ixazomib citrate minimally affects Rad51 or 53BP1 in SKOV3. (A) RT–qPCR of HR genes (BRCA1, BRCA2, RAD51, PALB2) in SKOV3 cells treated with ixazomib citrate (85 nM) for 0–24 h. mRNA levels were normalized to ACTB. (B) Immunoblot analysis of RAD51 protein expression in SKOV3 cells treated with ixazomib citrate (85 nM) for 0–24 h; β-actin served as the loading control. (C) Representative immunofluorescence images of 53BP1 foci (red) in SKOV3 cells after 24 h treatment with vehicle, olaparib (10 μM), ixazomib citrate (85 nM), or their combination. Nuclei were counterstained with DAPI (blue); scale bar, 20 μm. (D) Quantification of cells with >3 53BP1 foci per nucleus, calculated from ≥50 cells per condition. (E) Immunoblot analysis of 53BP1 protein levels in SKOV3 cells under the indicated treatments; β-actin served as the loading control. Data mean ± SD, n = 3; one-way ANOVA. [file Image2.jpeg]

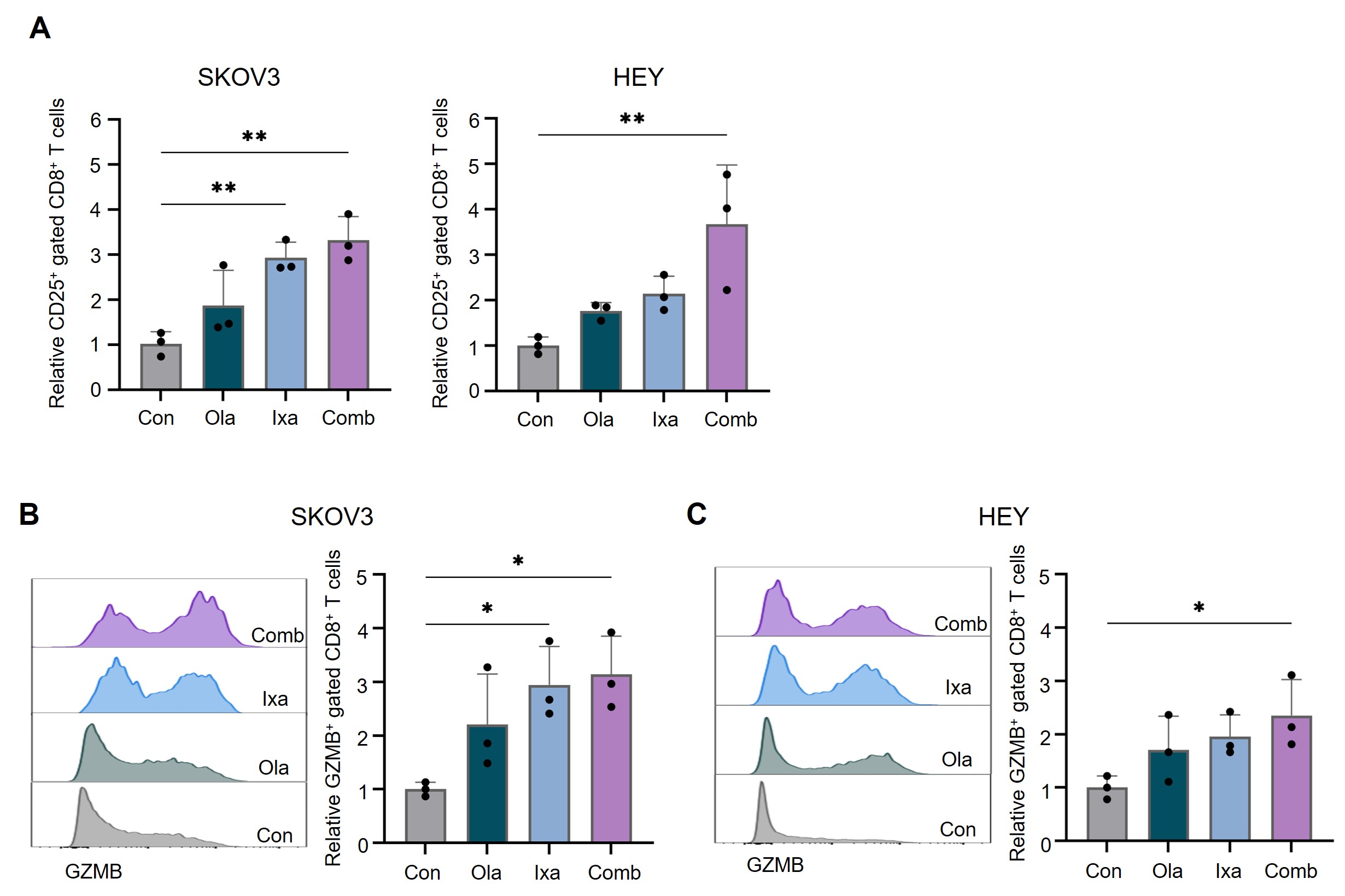

Supplement: Supplementary Figure 3 — Combined treatment enhances CD8+ T-cell activation. (A) Relative percentage of CD25+ cells among CD8+ T cells after co-culture with drug-pretreated SKOV3 or HEY cells, normalized to the control group. (B, C) Representative histograms (B) and quantification (C) of granzyme B (GZMB) expression in CD8+ T cells following co-culture with drug-pretreated SKOV3 or HEY cells. Data mean ± SD, n = 3; one-way ANOVA. *p < 0.05, **p < 0.01. [file Image3.jpeg]
